# Complete Genome Sequence of “*Candidatus* Nanosynbacter” Strain HMT-348_TM7c-JB, a Member of *Saccharibacteria* Clade G1

**DOI:** 10.1128/mra.00023-22

**Published:** 2022-04-11

**Authors:** Jonathon L. Baker

**Affiliations:** a Genomic Medicine Group, J. Craig Venter Institute, La Jolla, California, USA; b Department of Pediatrics, U.C. San Diego School of Medicine, La Jolla, California, USA; Indiana University, Bloomington

## Abstract

*Saccharibacteria* are abundant and diverse members of the human oral microbiome; however, they are poorly understood and appear to exhibit an epibiont/parasitic lifestyle dependent on host bacteria. Here, a complete metagenome-assembled genome (MAG) sequence of an organism from *Saccharibacteria* clade G1 human microbial taxon (HMT) 348 is reported, strain HMT-348_TM7c-JB.

## ANNOUNCEMENT

*Saccharibacteria* have a small cell size, reduced genomes, and appear to have an epiparasitic lifestyle, dependent on host bacteria (1–3). *Saccharibacteria* are common constituents of the oral microbiota, and although they have been correlated with inflammation and disease, their relationship to human health and their overall physiology and lifestyle are poorly understood (4–7). There are at least 6 major clades of oral *Saccharibacteria*, with the clade G1 group, human microbial taxon (HMT) 348, being one of the most abundant *Saccharibacteria* groups detected in supragingival and subgingival plaque, and on the buccal mucosa (6, 8, 9). Draft genomes of HMT-348 have been binned out of saliva and plaque sequencing libraries (4, 9) or assembled as single-cell amplified genomes (SAGs) (10, 11). Although one study was able to isolate an HMT-348 organism along with a putative host species (10), at the time of this work, no complete genomes of HMT-348 were available. Obtaining complete genomes is of special importance for *Saccharibacteria*, as they frequently lack the “essential” core genes and pathways that are used for determining draft genome completeness (3, 8). Furthermore, complete *Saccharibacteria* genomes are also helpful in guiding the isolation, culture, and subsequent study of *Saccharibacteria*, which has proven to be an immense challenge (12). In this study, Nanopore sequencing was used to obtain the complete genome sequence of a *Saccharibacteria* HMT-348 organism, HMT-348_TM7c-JB.

The draft assembly of HMT-348_TM7c-JB, “*Candidatus* Nanosynbacter sp.” isolate JCVI_32_bin.19, was reported in 2021, obtained from human saliva in Los Angeles, CA, USA, and fragmented into 7 contigs (4). From the same saliva sample used to obtain the original draft genome sequence, high-molecular-weight genomic DNA was extracted using a phenol:chloroform-based protocol (13) and examined for purity, size, and concentration using a TapeStation instrument (Agilent Technologies). The DNA was not sheared or size selected. A long-read library was prepared using a ligation sequencing kit (Oxford Nanopore Technologies) and sequenced on a GridION using an R9.4.1 flow cell (Oxford Nanopore Technologies). Base calling, quality control, error correction, and adapter trimming were performed using Guppy v4.0.11/MinKNOW v20.06.9 (Oxford Nanopore Technologies), resulting in 9.7 million reads (*N*_50_, 6,360 bp). Human reads were removed using minimap2 v2.17-r941 (14), and the remaining long reads were assembled using meta-Flye v2.9-b1768 (15). Among the contigs generated in the Flye metagenomic assembly, a circular 841,116-bp fragment was obtained with >99% average nucleotide identity (ANI; determined using Anvi’o [16]) to “*Candidatus* Nanosynbacter sp.” isolate JCVI_32_bin.19. Illumina reads from the original short-read library (used to generate the sequence for “*Candidatus* Nanosynbacter sp.” isolate JCVI_32_bin.19) were mapped to the Flye contig using BWA-MEM v0.7.17-r1188 (17) and used with Polypolish v0.4.3 to remove errors remaining in the circular Flye draft genome. HMT-348_TM7c-JB was annotated using the NCBI Prokaryotic Genome Annotation Pipeline v5.1. Circulator v1.5.5 (18) was used to rotate the genome start to *dnaA*. For all software, default parameters were used unless otherwise noted. The resulting chromosome was 841,302 bp long with a GC content of 38.21%, and is predicted to encode 852 genes. This complete metagenome-assembled genome (MAG) will provide valuable information regarding the lifestyle and evolution of “*Candidatus* Nanosynbacter sp.” HMT-348.

### Data availability.

The complete genome sequence of HMT-348_TM7c-JB is available via GenBank under the accession number CP090820.1. The BioProject accession number for the genome is PRJNA624185. The short reads used to polish the draft genome are available in the Sequence Read Archive (SRA) database under the accession number SRX4318835, and the long reads used to generate the draft assembly are available under SRA accession number SRX13639916.
